# Increasing health policy and systems research capacity in low- and middle-income countries: results from a bibliometric analysis

**DOI:** 10.1186/s12961-017-0229-1

**Published:** 2017-07-28

**Authors:** Krista M. English, Babak Pourbohloul

**Affiliations:** 1 Complexity Science Lab, School of Population & Public Health, University of British Columbia, Vancouver, British Columbia Canada; 2 Institute of Resources, Environment and Sustainability, University of British Columbia, Vancouver, British Columbia Canada

**Keywords:** Health policy, Systems research, Low- and middle-income countries, Knowledge production, Capacity-building

## Abstract

**Background:**

For 20 years, substantial effort has been devoted to catalyse health policy and systems research (HPSR) to support vulnerable populations and resource-constrained regions through increased funding, institutional capacity-building and knowledge production; yet, participation from low- and middle-income countries (LMICs) is underrepresented in HPSR knowledge production.

**Methods:**

A bibliometric analysis of HPSR literature was conducted using a high-level keyword search. Health policy and/or health systems literature with a topic relevant to LMICs and whose lead author’s affiliation is in an LMIC were included for analysis. The trends in knowledge production from 1990 to 2015 were examined to understand how investment in HPSR benefits those it means to serve.

**Results:**

The total number of papers published in PubMed increases each year. HPSR publications represent approximately 10% of these publications, but this percentage is increasing at a greater rate than PubMed publications overall and the discipline is holding this momentum. HPSR publications with topics relevant to LMICs and an LMIC-affiliated lead authors (specifically from low-income countries) are increasing at a greater rate than any other category within the scope of this analysis.

**Conclusions:**

While the absolute number of publications remains low, lead authors from an LMIC have participated exponentially in the life and biomedical sciences (PubMed) since the early 2000s. HPSR publications with a topic relevant to LMICs and an LMIC lead author continue to increase at a greater rate than the life and biomedical science topics in general. This correlation is likely due to increased capacity for research within LMICs and the support for publications surrounding large HPSR initiatives. These findings provide strong evidence that continued support is key to the longevity and enhancement of HPSR toward its mandate.

**Electronic supplementary material:**

The online version of this article (doi:10.1186/s12961-017-0229-1) contains supplementary material, which is available to authorized users.

## Background

A broad, interdisciplinary and applied field bringing together economics, sociology, anthropology, political science, public health and epidemiology, health policy and systems research (HPSR) is centred around how “*health systems and policies shape and are shaped, both by each other and by the broader determinants of health*” [1]. HPSR thus has much to contribute to health systems strengthening and, in turn, to the improvement of health outcomes, with the field defined more in terms of the questions it addresses rather than being rigidly constrained by methodological or disciplinary constraints [1, 2].

The field of HPSR has evolved significantly over the past few decades. The success of four Global Symposia on Health Systems Research, and the establishment of the membership society Health Systems Global reflect the crystallisation and growth of an HPSR research community. On the other hand, the development of the WHO Strategy on HPSR, the recognition of HPSR in the World Health Report 2013 and the establishment of the Health Policy Leadership Initiative bringing together policymakers to identify HPSR research priorities underscore a greatly increased level of interest in HPSR among policy and decision-makers [1].

This paper seeks to take stock of the evolution of HPSR production over these past decades through a bibliometric analysis of HPSR publications for the period 1990–2015. Given the applied, context-sensitive nature of HPSR knowledge, it is important that HPSR produced is relevant to specific country settings, this is particularly true for low- and middle-income countries (LMICs), where health systems strengthening efforts have often not been adequately informed by locally relevant research. In light of this, we specifically examined HPSR produced in relation to LMICs. In addition to being indicative of the growth and evolution of the field, trends in research production also serve as a useful proxy for research capacity. Given the need to understand the evolution of HPSR research capacity in LMICs, we examined the production of HPSR in LMICs. Finally, it is important to know the distribution of research production by topical areas within HPSR. This has been achieved through an examination of HPSR research production in terms of the six building blocks (BBs) of the health system as put forth by WHO.

This is a particularly opportune moment for analysing the growth and evolution of HPSR. The importance of health systems strengthening and appropriate research to inform these efforts has been brought to the fore by crises including the Ebola epidemic [1]. At a broader level, the Sustainable Development Goals (SDGs) have brought attention to the importance of working across sectors and taking an integrated view of development. HPSR is an applied field, drawing on several disciplines, and thus has much potential to contribute towards the achievement of the SDGs [1]. Finally, it is a little over 20 years ago that the decision was taken to establish an entity dedicated to HPSR, which took shape in 1999 as the Alliance for Health Policy and Systems Research, an international partnership housed within WHO that has had a crucial role in catalysing this evolution and building the field of HPSR [3].

The paper is divided into three sections, namely the methods used to carry out the bibliometric analysis and a presentation of the findings, followed by a discussion and conclusion.

## Methods

For the purpose of this study, HPSR is defined as research on the health system functions of regulation, organisation, financing and delivery of services, as well as broader determinants (such as social and economic policies directly affecting the health system) [2]. It focuses primarily upon the more upstream aspects of health, organisations, policies and programmes, but does not address clinical management of patients or basic scientific research [2, 4].

### Bibliometric analysis

At this milestone in HPSR history, evaluation and reflection of the contribution is captured through a variety of quantitative analyses. A bibliometric analysis is one type of quantitative analysis used to examine the production of academic literature over time. It is used to assess the impact of a field, researcher(s), or a paper over time. These methods are intended to infer a correlation between impact and influence and participation and connectedness in the published literature. Participation is measured by frequency of publication, while connectedness is measured by co-authorship (English K, Ghaffar A, Shroff Z, Pourbohloul B, Health Policy and Systems Research Collaboration Pathways: Lessons from a Network Science Analysis. In Review, submitted to Health Research Policy and Systems, 2017).

### Databases

Eleven databases among the University of British Columbia Library Catalogue demonstrated an intellectual contribution to the field of HPSR, under traditional and emerging subject areas (Additional file 1). Since many of these databases are not traditionally related to health policy, a preliminary review was conducted of each database using high-level keywords to identify the proportion of potentially relevant papers.

Web of Science is the largest database and has approximately 50% more publications than PubMed [5, 6]. PubMed is the next largest but has approximately twice as many health policy publications as Web of Science. PubMed is also a free search engine accessing primarily the MEDLINE database of references and abstracts on life and biomedical sciences, and biomedical topics. In the remaining databases, the percentage of relevant publications may be quite high but the absolute number is very low. Therefore, PubMed was selected as the database to analyse HPSR.

### Search strategy

There are five components for the search strategy. A high-level search strategy was defined, suitable for the multi-decade timescale of a bibliometric and network analysis. Given that keywords, terms and topics may trend over time, the high-level keywords selected were ubiquitous and consistent.

### Defining HPSR

To ensure inclusivity of publications related to HPSR, a high-level keyword search strategy was applied. This strategy assumes that publications related to HPSR would, at the very least, have the words (health AND policy) OR “health system(s)” somewhere within the entire text of the publication. Once these publications were identified, additional keywords could be included to refine the definition.

The syntax (health AND policy) implies both terms are required in a single paper for inclusion. Alternatively, if a paper had the specific term “health system*” either independently or in combination with (health AND policy), it was also eligible for inclusion. Applying the asterisk implies all potential variants extending from the expression shall also be included, such as “health systems”.

In the literature, disciplinary inclusion can be broad while exclusion is better defined. PubMed includes a defined set of filters to identify specific topics related to clinical queries and medical genetics [7]. The exclusion criteria can be applied to the search strategy using the Boolean operator, “NOT” thereby removing the irrelevant clinical literature [8].

The species filter was applied to restrict the results to human studies [9].

### Relevance to LMICs

Given the context sensitivity of the findings of HPSR, it is important that efforts to strengthen health systems in LMICs be informed by research that is produced specific to particular contextual settings.

This analysis thus identifies the collection of papers with its main topic focused on an issue relevant to a LMIC (referred to in the figures as “LMIC Topic”). The title and abstract sections, denoted by the tag “Title/Abstract [TIAB]”, are intended to most concisely describe the main focus and purpose of a paper. Therefore, these papers can be efficiently identified by limiting the search to the list of 135 low-income countries (LICs), lower middle-income countries (Lower-MIC) and upper middle-income countries (Upper-MIC) and synonyms for ‘developing country’ that appear in the title and abstract [10]. This strategy is used in combination with the keyword search strategy.

### LMIC authors

These analyses are designed to help our understanding of the extent to which LMICs participate meaningfully in the HPSR that is meant to support decision-making capacity in their countries. Between 1998 and 2014, only the first author’s affiliation was included in PubMed [11]. Identifying lead authors from LMICs is one means to determine participation; frequency of publication and connectedness of their co-authorship networks over time are used as the metrics.

To identify authors from LMICs, a combination of each of the 135 LMICs was used with PubMed’s advanced search field builder [Affiliation]. An “LMIC Author” was defined as a first author whose institutional affiliation/address included an LMIC, this address was used as a proxy for country of residence.

### List and classification of countries

For the fiscal year 2016, the World Bank has identified 135 LMICs and 80 high-income countries (HICs). LMICs generally refers to the three sub-classifications that represent LMICs, inclusive of all LICs, Lower-MICs and Upper-MICs [12]. We have altered the syntax to reduce confusion between lower middle-income countries and low- or middle-income countries, both of which are typically abbreviated as LMICs. In addition to the individual name of each country, the strategy includes synonyms for developing countries, [dev countr*] (Additional file 1). The inclusion of these terms captures papers that may refer to developing countries more generally as a main topic (in the title and or abstract) without listing the name of the country explicitly.

### Analysis over time

A publication date filter was used to restrict the studies to each and all years inclusive of January 1, 1990, to December 31, 2015. The range of years is meant to span beyond the inception of the Alliance for Health Policy and Systems Research to identify a baseline.

## Results

**Fig. 1:**
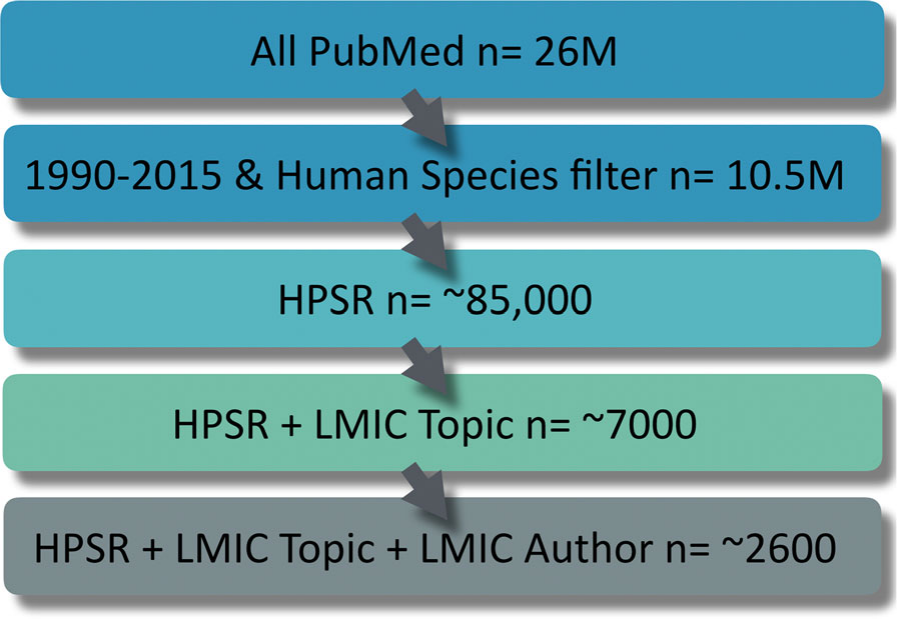
Number of Publications

PubMed is comprised of more than 26 million papers; almost 16.7 million of which were published between January 1, 1990, and December 31, 2015, and 10.5 million remain for the same period, once the human species filter is applied  (Fig. 1). This latter group represents the baseline for this analysis and is used to show the general increase in publications for the specified period.

Each individual component was examined independently to understand its contribution to any topic in PubMed over time before moving on to the cumulative analysis. The number of publications per individual component is higher than the compound effect.

### Frequency of publication per independent component

**Fig. 2:**
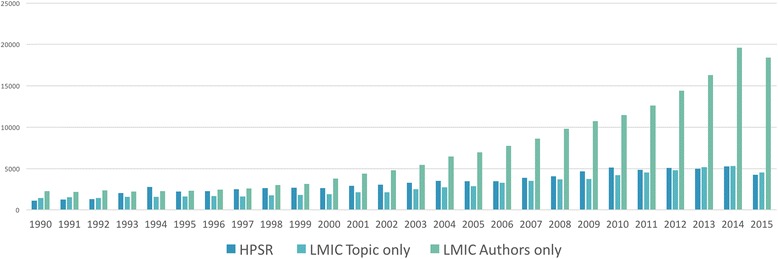
Contribution of each independent component among all life and biomedical sciences in PubMed over time

Among all publications in PubMed, those that focus only on HPSR (blue bars) have been increasing slowly and steadily since 1990. Publications that focus only on an issue relevant to an LMIC (and may or may not also focus on HPSR) (teal bars) typically lag behind HPSR publications (Fig. 2). In contrast to these modest progressions, first authors from LMICs have participated exponentially in the life and biomedical sciences (PubMed) since the early 2000s. In the early 2000s, first authors from LMICs published about twice as many papers in PubMed than the number of publication that focused on a topic relevant to LMICs, this rate has since steadily increased and in 2015 – it was four times as many publications. Given that there is one lead author per publication in PubMed, the increasing share among LMIC lead authors since 2000, is greater than that of PubMed overall.

Since we know publications overall are increasing, we would like to understand whether the pace of publication among HPSR papers, with a topic relevant to LMICs and lead authorship from an LMIC is underperforming, on par, or outpacing life and biomedical sciences in general.

Subsequent analysis includes the cumulative effect of HPSR literature combined with LMIC Topic and LMIC Author. It can be assumed that approximately 2/3 (n ~ 4400) of HPSR publications with a topic relevant to LMICs have lead authors affiliated with HICs. Some analysis identifies the absolute number of publications, while others show normalised slopes. Certain features are more visually apparent when the data is normalised to 1 in the most productive year per category. It allows for a fair comparison between data of different scales (different denominators).

All publications in PubMed (limited to human species) have increased from just over 260,000 per year in 1990 to the maximum thus far of approximately 615,000 in 2014. In figure format, subsequent categories would be almost invisible given the scale that would be required for the vertical axis. In most figures, 2015 generally appears to have fewer publications but this is due to a lag between the publication date in some journals and their appearance (publication date) in PubMed. Given time, this year will continue the upward trend seen in all previous years.

### Number of publications

**Fig. 3:**
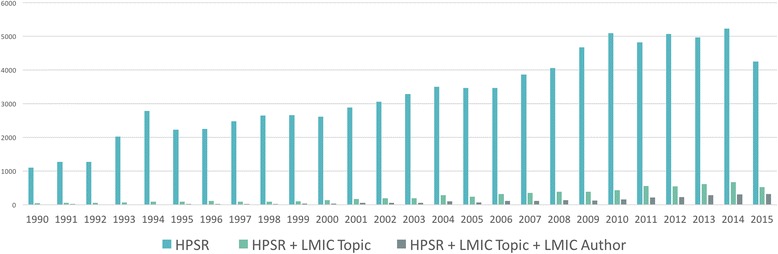
Absolute number of health policy and systems research publications in PubMed from 1990 to 2015

Figure 3 demonstrates the contribution, in terms of absolute number, of HPSR and its sub-categories over time. All three categories have been increasing since 1990. HPSR publications consistently comprise approximately 10% of all publications in PubMed. These are followed by HPSR publications that have a main topic focused on any LMIC and the further sub-set of the aforementioned, which also have a first author whose main affiliation is in an LMIC. The last category provides a relatively small contribution (0.004%–0.067% depending on the year) to the body of knowledge in PubMed overall.

It is worth noting the increase in HPSR publications in the mid-1990’s and again a significant ramp up around the time of the First Global HSR Symposium in 2010.

### Normalised comparison of HPSR literature to all PubMed 1990–2015

**Fig. 4:**
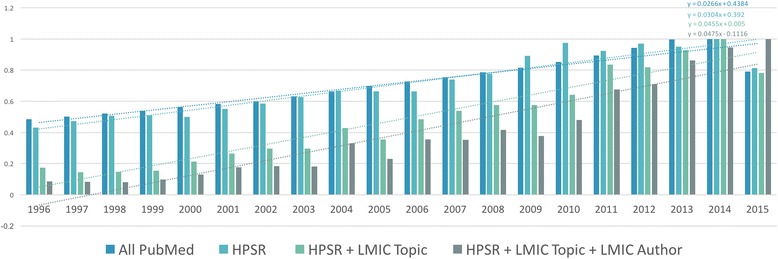
Normalised comparison of each incremental category per year

The normalisation in Fig. 4 clarifies the significant and increasing contribution of the category HPSR + LMIC Topic + LMIC Author compared to Fig. 3, in recent years. Specifically, in 1990–2015, the trend in HPSR production, as measured by the high-level keywords, increased at a greater rate than publications in PubMed overall. HPSR publications with a topic relevant to LMICs also increased at a greater rate than the aforementioned. During this period, HPSR publications with a topic relevant to LMICs and a lead author from an LMIC is clearly an emerging area and is on pace with the previous category. In the early 2000s, there was a visible boost in this latter category to where it began to outpace all other categories in the last decade. This increase in HPSR is likely due, in part, to the fact that, around the same time, authors from LMICs had an increased contribution to life and biomedical sciences in general (Fig. 2). This overall increase may be the result of increased capacity in LMICs as per the intention of the Millennium Development Goals and/or many other development programmes, including the Alliance for Health Policy and Systems Research.

**Fig. 5:**
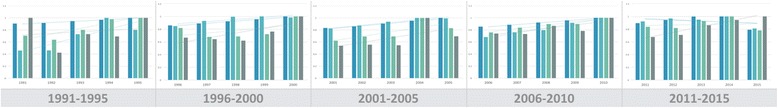
Normalised comparison of the slopes per 5-year intervals

The best linear fit of each time series is identified by the slope of that line (i.e. the coefficient *x* in the equations) (Fig. 4). Comparatively speaking, the higher the coefficient of *x* in the equation, the steeper the angle of the slope and the greater the increase in contribution to the discipline over time. The slope of the linear fit is important as it correlates to the amount of HPSR knowledge production, participation and institutional capacity. The data implies that the increase in publication frequency in HPSR literature from 1990 to 2015 outpaced life and biomedical sciences in general (PubMed), and that the discipline is holding its momentum. The slope of sub-ranges of years may also be reviewed to understand different patterns of change over time (Fig. 5). For example, there have been some very progressive contributions by LMICs in the last decade, where the slope would be much steeper than over the full 26-year period.

The slope of all HPSR papers with a topic focused on LMICs is slightly steeper than the HPSR papers in general. This is an effect of the cumulative combination, meaning that the number of HPSR papers being published with a focus on LMICs is increasing at a greater rate than HPSR papers in general. The steepest slope is among HPSR papers with a topic focused on LMICs and with a first author affiliated in an LMIC. While the absolute number remains low, the percentage of contribution is increasing at a slightly greater pace than the other categories over time.

While the largest increase in production of HPSR knowledge is among LMIC first authors writing about LMIC-relevant topics (grey bars), we acknowledge that correlation does not imply causation. Nonetheless, this outcome may be indicative of the positive effect of the ongoing effort to ensure increased funding and institutional capacity-building, and that knowledge production continues to support vulnerable populations and resource-constrained settings. These results provide strong evidence to demonstrate that continued investment and evaluation will ensure success and meaningful inclusion of the regions that HPSR supports.

### Comparison of productivity per 5-year interval

During the early 1990s, publication in all PubMed (blue) and HPSR + LMIC Topic + LMIC Author (grey) followed a similar, and almost parallel, trajectory ﻿(Fig﻿. ﻿5)﻿. From 1996 to 2006, knowledge production among all categories was quite irregular, with no clear pattern. After 2006, all sub-categories began increasing and were highly productive.

Immediately following the inaugural HSR Global Symposium in 2010, there was a substantial increase in all categories of HPSR knowledge production. HPSR + LMIC Topic + LMIC Author (grey), is on trend to surpass the continuing increases in all other categories.

The most intriguing changes have occurred in the most recent years, whereby subsequent categories have begun to outpace the All PubMed (blue) group. The HPSR + LMIC Topic (green) and HPSR + LMIC Topic + LMIC Author (grey) have improved so significantly that All PubMed (blue) and HPSR (teal) appear to be declining in prominence relative to the former.

### Distribution and influence of BBs over time

HPSR focuses primarily upon policies, organisations and programmes, but does not address clinical management of patients or basic scientific research (for example, on cell or molecular structures). Heath systems have historically been viewed within a framework of six BBs, namely Health Financing, Health Workforce, Information and Evidence/Research, Leadership and Governance, Medical Products and Technologies and Service Delivery.

In the literature, disciplinary inclusion within a BB can be broad while exclusion is better defined, yet, in practice, the boundaries are often blurred (Additional file 1). An approach to understanding the field of HPSR is to analyse each of these components. At any given time, each of the six BBs is in different states of development and definition. Given this reality, the definition of some BBs is more challenging than for others. Additionally, attempting to compartmentalise the complexity of health systems into six discrete components may be an over-simplification.

Below, HPSR publications that focus on an LMIC Topic and any of the six BBs were examined over time. Given the blurred boundaries between the BB, a high-level definition was used, similar to the HPSR search strategy, to ensure inclusivity. The strategy included HPSR + LMIC Topic + BB. As in the previous case, subsequent keywords could be added to further refine each BB but may reduce the absolute number of publications eligible for inclusion to below a reasonable threshold for meaningful analysis.

#### HPSR publications with an LMIC topic by BB

**Fig. 6:**
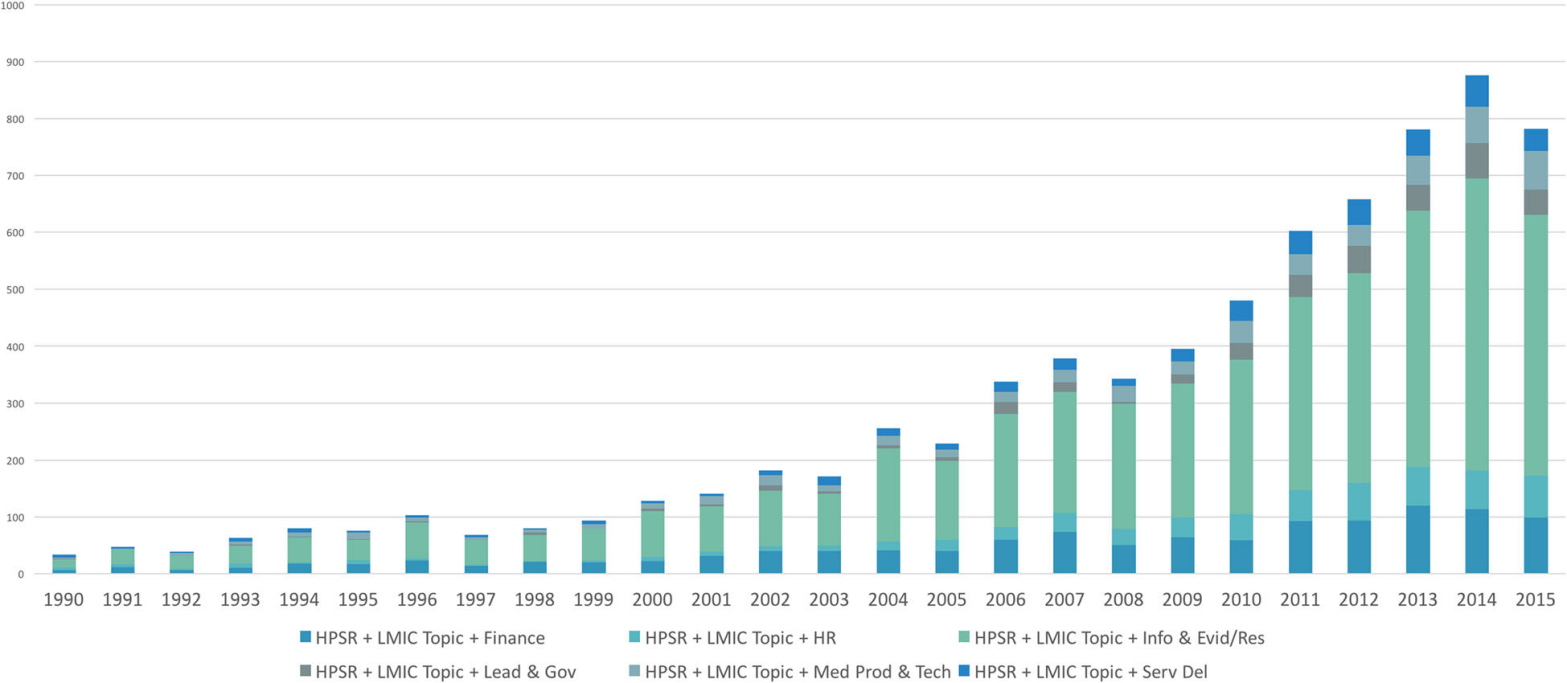
Health policy and systems research publications that focus on a low- and middle-income country topic sub-divided by building block over time

Topics related to the BBs (Fig. 6) referred to as Information and Evidence/Research represent almost 60% of the HPSR literature captured in the graph above. Research and evidence is a prominent and resilient tenant of HPSR. Its increasing prominence since the inception of HPSR demonstrates its importance to the discipline.

While attempts have been made to define HPSR by BB [13, 14, 15], the name of each BB has changed over the years and therefore carries a different meaning/context depending on the publication (Additional file 1) [4, 14, 16, 17]. There are inherent overlaps that make it impossible to disentangle the six BBs. In addition, there is no apparent benefit to the discipline in attempting to do so.

### Participation by country and income group

**Fig. 7:**
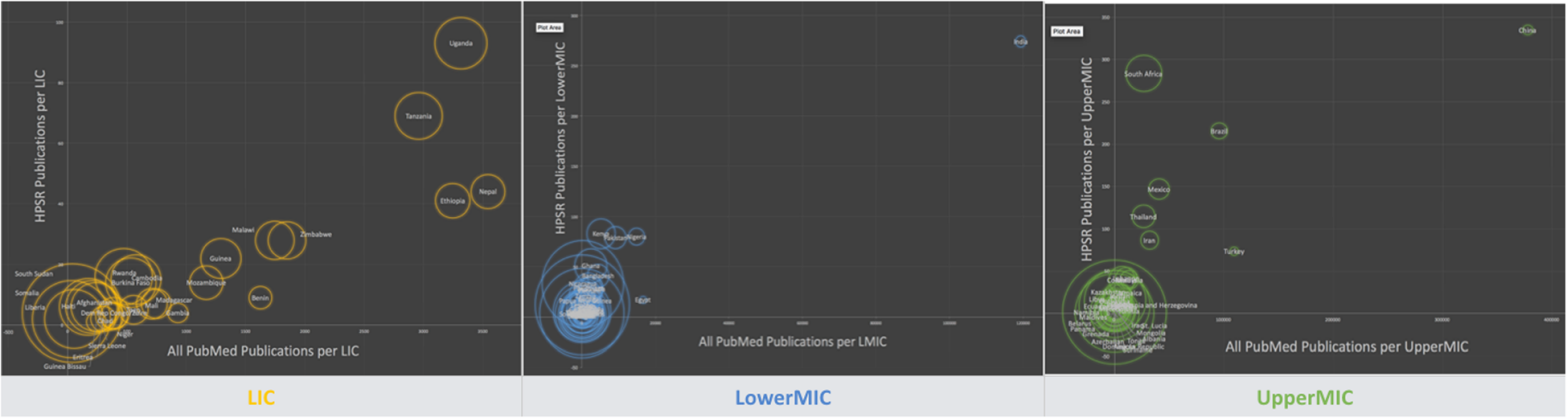
Comparison of contribution to health policy and systems research versus all PubMed publications by authors from each country per low- and middle-income country income group

**Fig. 8:**
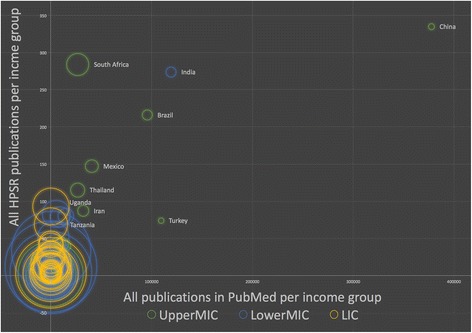
Relative comparison of contribution to health policy and systems research versus all PubMed publications by authors from each country per low- and middle-income country income group

Figure 7 demonstrates the contribution to HPSR by country and income group. Each circle represents a country and the colour represents the income group. The x-axis identifies the number of publications on any topic in PubMed between 1990 and 2015. The y-axis identifies the number of HPSR publications for the same period. In each of the three graphs, the scale is dramatically different, this becomes more apparent in the large combined graph (Fig. 8). The size of the circle is the percentage of HPSR publications of all PubMed publications as per the lead author’s national affiliation. Generally, the number of publications with lead authors from LMICs is fairly low, with the exception of a few outliers.

Larger circles have a higher percentage of HPSR publications. The large circles appearing closer to the origin (*O*) indicates that, while the absolute number of publications might be low, there is still a higher percentage of HPSR publications.

#### Percentage of all HPSR/PubMed per LMIC group

The main outliers among LICs (yellow circles) include Uganda, Tanzania, Ethiopia and Nepal (Fig. 7). LIC contributions appear to be the most horizontally diffused in the Cartesian plane. India outperforms the next closest Lower-MIC (blue circles), with 7-fold Egypt’s number of publications in all PubMed and greater than 3-fold the number of HPSR publications by Kenya. Among Upper-MIC (green circles), China publishes most frequently in both categories but the proportion of HPSR is low relative to the overall contribution to PubMed. South Africa, Mexico, Brazil, Iran and Turkey are also outliers with a stronger contribution to HPSR literature (as per the more vertical spread along the y-axis).

The perception of contribution may change once the graphs are combined and scaled. Relative to other income groups, the concentration of LICs is now diffused vertically. While the absolute numbers are lower, LICs contribute a significant proportion of their publications to HPSR (bigger circles distributed vertically along the y-axis).

## Percentage of HPSR/PubMed per income group

### Frequency of individual LMIC author publication and citation

Among the approximately 7000 HPSR publications with a topic relevant to LMIC (HPSR + LMIC Topic), there were 15,701 individual authors, 6940 of which are from LMICs (Fig. ﻿8). Of these, 82 had 10 or more publications, 59% of which were affiliated in an LMIC. While only six authors had 20 or more publications, four of these authors were from LMICs.

A total of 118 authors have had publications cited 100 times or more, 36% of these are affiliated in an LMIC; 19 authors have been cited 200 times or more and all were from Upper-MIC or HICs (58%); four authors have been cited more than 300 times, 75% were from HICs.

The figures below feature each of the LMIC country income groups and the normalised number of publications by lead authors from LMICs. The normalised comparison is advantageous over the frequency as countries with many publications make those with fewer appear visually insignificant. While there are 135 LMICs in total, the graphs include approximately half this number. If there were fewer than 500 publications per country from 1990 to 2015, an annual breakdown was not available. Therefore, this is a compilation of all countries within each income group that produced a total of more than 500 publications over the full duration of the study.

### LICs

#### Frequency of publication by LIC author affiliation

**Fig. 9:**
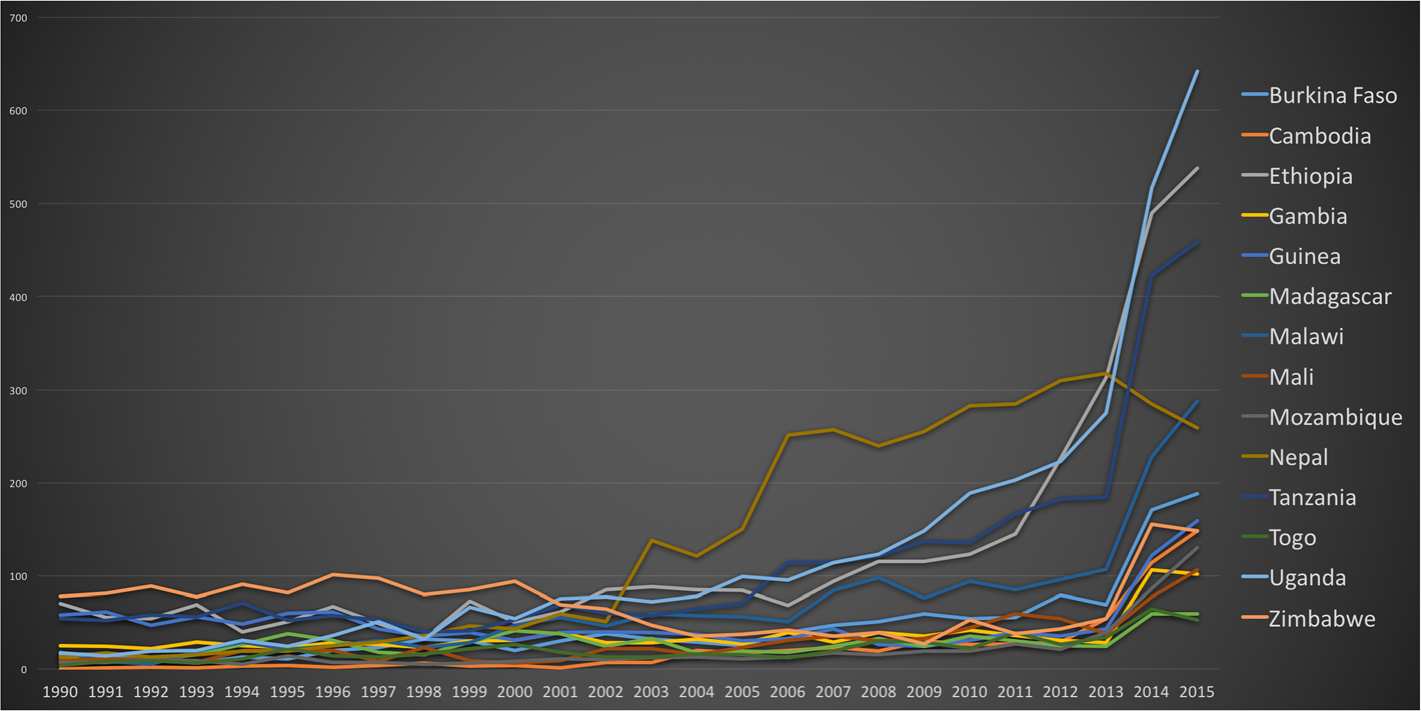
Frequency of publication by low-income country author affiliation

Figure 9 demonstrates the absolute number of HPSR + LMIC Topic publications by lead authors from LICs. The year 2002 was a turning point for publication frequency, both in terms of absolute number and normalisation. All countries begin to elevate from the x-axis at this time. In the normalised figures, the thick blue line represents the baseline of all PubMed publications. Prior to 2002, authors from Zimbabwe published most frequently among all LICs and after this point their publications dropped to among the lowest. At the same time, authors from Nepal, Uganda, Tanzania and Ethiopia increased their publication frequency to lead among LICs. By 2013, Malawi, Burkina Faso, Zimbabwe, Guinea, Cambodia, Mozambique and Mali saw a slight increase in publication by authors from their countries. Gambia, Togo and Madagascar saw little to no increase in publication frequency for the duration of the study period.

#### Normalised comparison of LIC authored publications

**Fig. 10:**
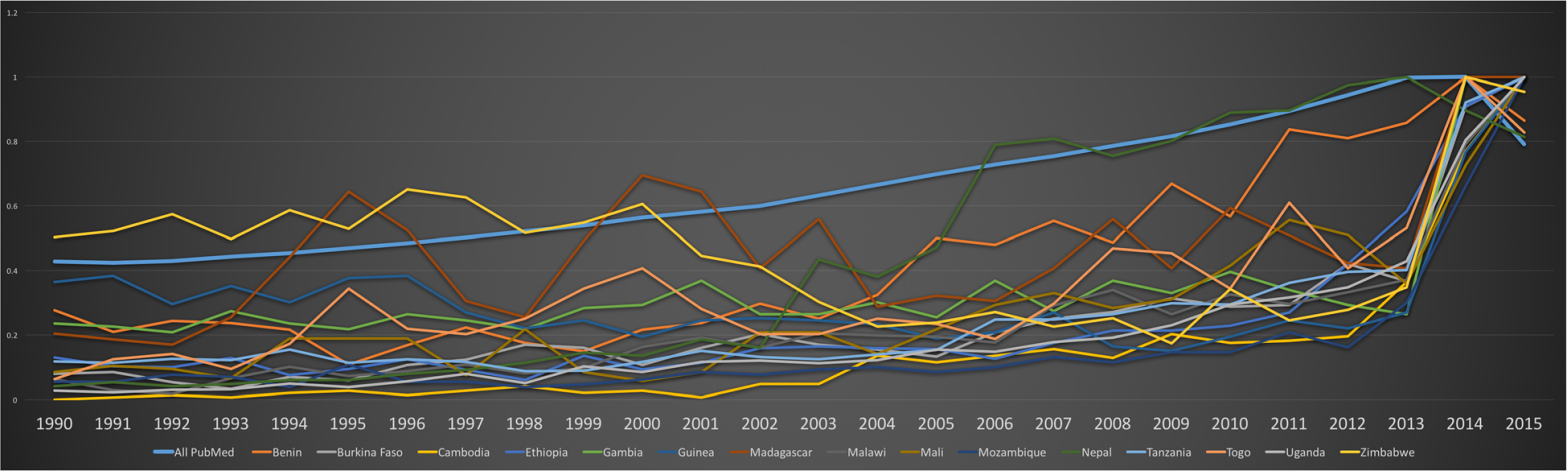
Normalised comparison of publications by first authors from low-income countries

Figure 10 shows a normalised comparison of LIC authored publications. Frequency figures are not depicted in subsequent income groups. The disproportionately large contribution of India (Lower-MIC) and China (Upper-MIC) appear to diminish the contribution of all other countries rendering them practically indistinguishable.

### Lower-MICs

Authors from India produced almost 10-fold that of the authors from the next most frequently published Lower-MIC. Nigeria and Egypt were a distant second and third, respectively, until 2009, at which point they switch places. As in LICs, there appeared to be a shift around 2002, whereby Pakistan separated from the other countries to reach the fourth place. Kenya and Bangladesh emerged to follow by the mid-2000s.

#### Normalised comparison of Lower-MIC authored publications

**Fig. 11:**
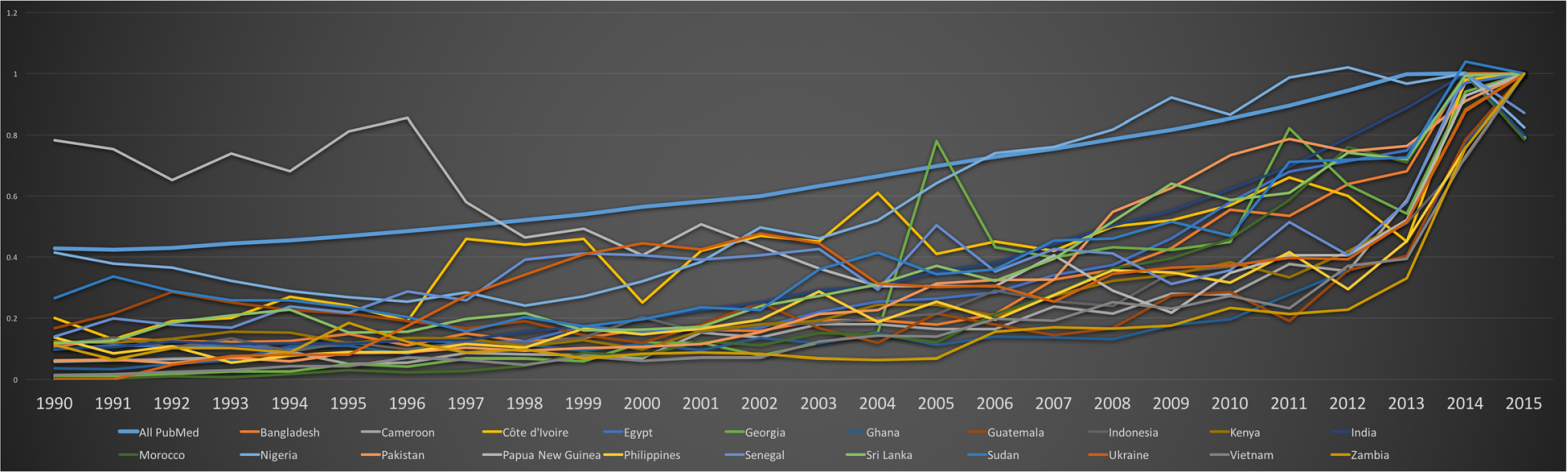
Normalised comparison of publications by first authors from lower-middle income countries

In the normalised Fig. 11 above, countries begin to elevate from the x-axis in the late 1990s. Papua New Guinea is clearly outpacing PubMed and other Lower-MICs until the late 1990s. Authors from Côte d'Ivoire, Senegal, Ukraine and Nigeria lead Lower-MIC publications around the turn of the century. Georgia, Pakistan and India show improved proportionality after the mid-2000s, keeping pace with the aforementioned countries.

### Upper-MICs

Similar to India among Lower-MICs, authors from China significantly out-produced all other authors from Upper-MICs across the study period, with the trend being overt in 2000. In 1990, Mexico was a distant second behind China. Authors from Mexico published consistently, with a slight increase over all years and holding the fifth place among all Upper-MIC. Around the year 2000, authors from Turkey and Brazil contributed to a distant second and third place, respectively, before switching in 2010, when Brazilian authors publish more frequently than those from Turkey. In terms of frequency of publication, the top countries were consistent across all years.

#### Normalised comparison of Upper-MIC authored publications

**Fig. 12:**
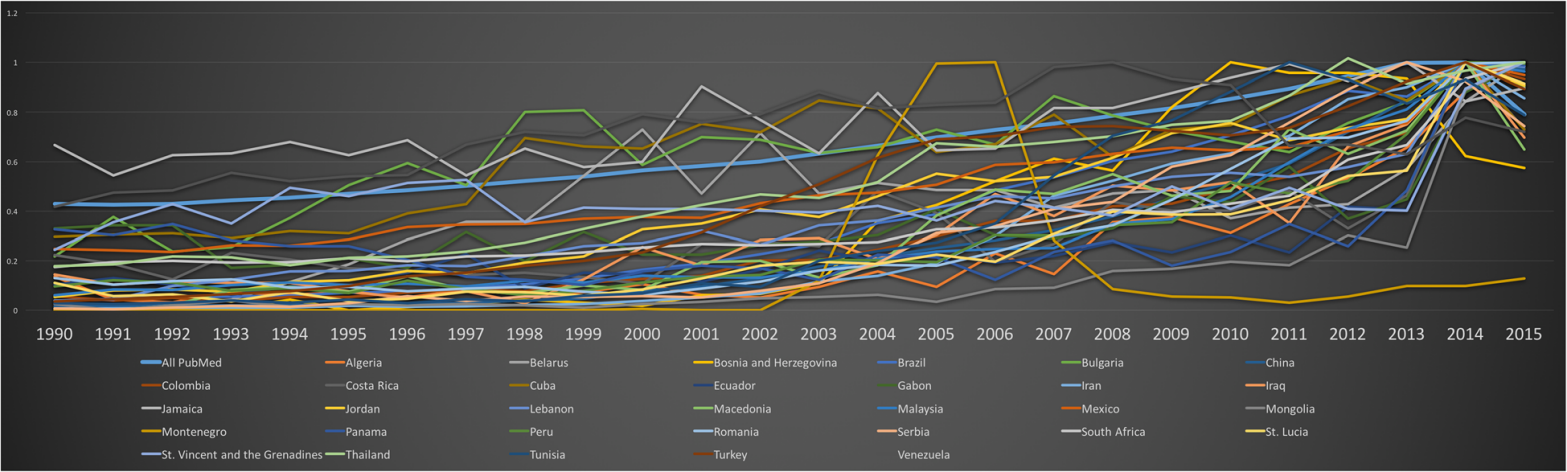
Normalised comparison of publications by first authors from upper-middle income countries

Authors from Venezuela, Jamaica, Cuba and Bulgaria all typically outpaced PubMed overall (Fig. 12). This characteristic was generally less common among individual countries within the LICs and Lower-MICs.

### Evolution of HPSR themes

There was a distinct difference between the Medical Subject Headings (MeSH) terms assigned and keywords selected for a paper by the authors.

The National Library of Medicine MeSH are a controlled vocabulary of biomedical terms used to describe the subject of each journal article in MEDLINE. Skilled subject analysts examine journal articles and assign the most applicable MeSH terms – typically 10–12. Applying the MeSH vocabulary ensures that articles are uniformly indexed by subject, regardless of the author’s suggested keywords [17]. Among HPSR publications, MeSH terms identify the species in every paper, the sex and age group of the population under study are also given priority.

The frequency of author-assigned keywords is significantly lower and less standardised, but arguably more representative of the papers’ topic. Author-assigned keywords among HPSR publications focus on the topic of the paper, with little emphasis on demographic information unless it pertains to the socioeconomic status as is relevant to LMICs.

This analysis examines MeSH terms because the vast majority (approximately 6/7 papers) do not have any author-assigned keywords from 2001 to 2011. The reason for this is unclear, but it is possible that PubMed did not require that bibliometric field during that period. We welcome future analysis to identify interesting features about trends in HPSR topics published over time. Please see Additional file 1 for a comparison of terms available.

The resulting terms and themes are significant to understanding the discipline as they were identified from the high-level keyword search used to capture the HPSR literature. The figures below demonstrate the dynamic trends in important MeSH themes over time.

#### Top 10 MeSH terms per year

**Fig. 13:**
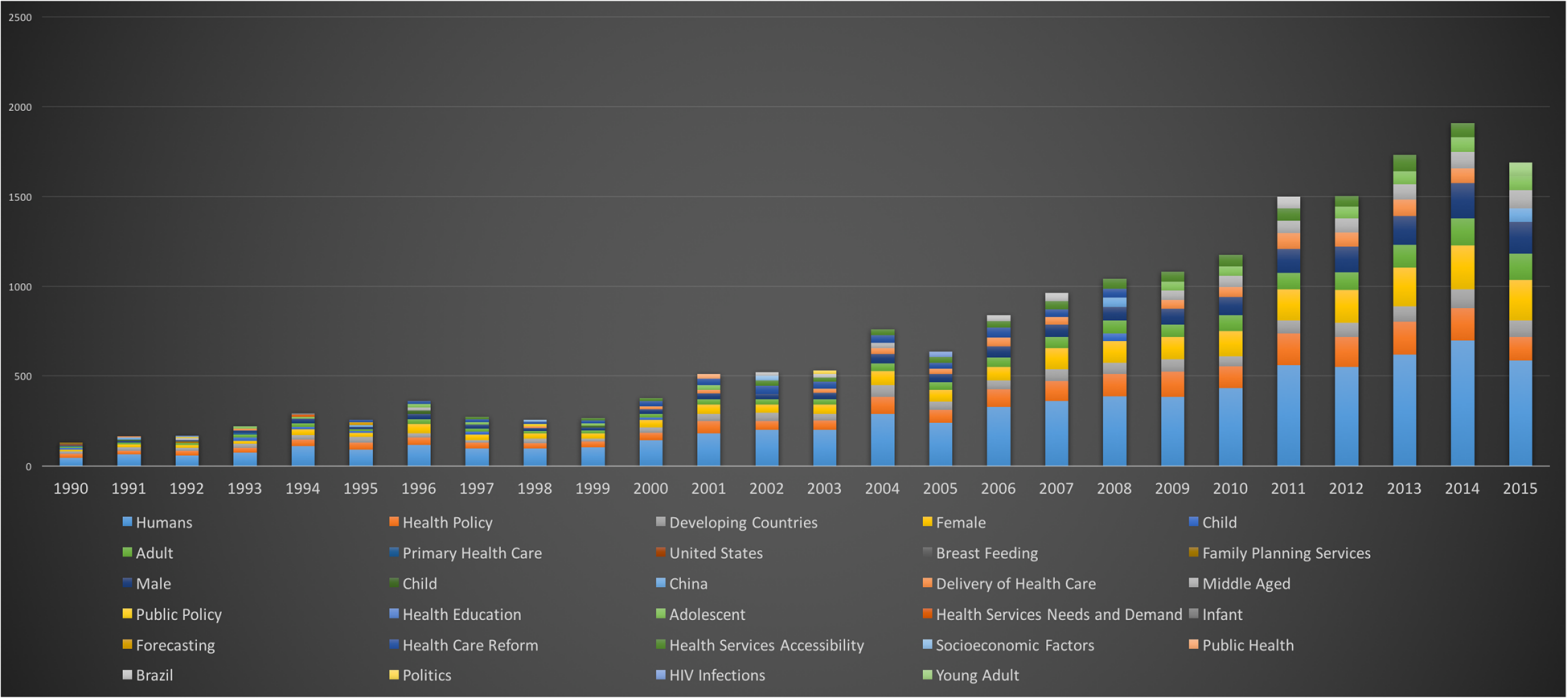
Top 10 MeSH terms assigned to health policy and systems research + low- and middle-income country topic publications per year

Overall, 75,704 MeSH terms were assigned to 7009 HPSR papers with a topic relevant to LMICs. Using the top 10 assigned terms for each publication year, we can note the changing trends in MeSH assignment (Fig. 13). While approximately the first five terms were consistently assigned, subsequent terms demonstrate the shifts in policy interest.

#### Top 10 MeSH terms per year – excluding demographics and geography

**Fig. 14:**
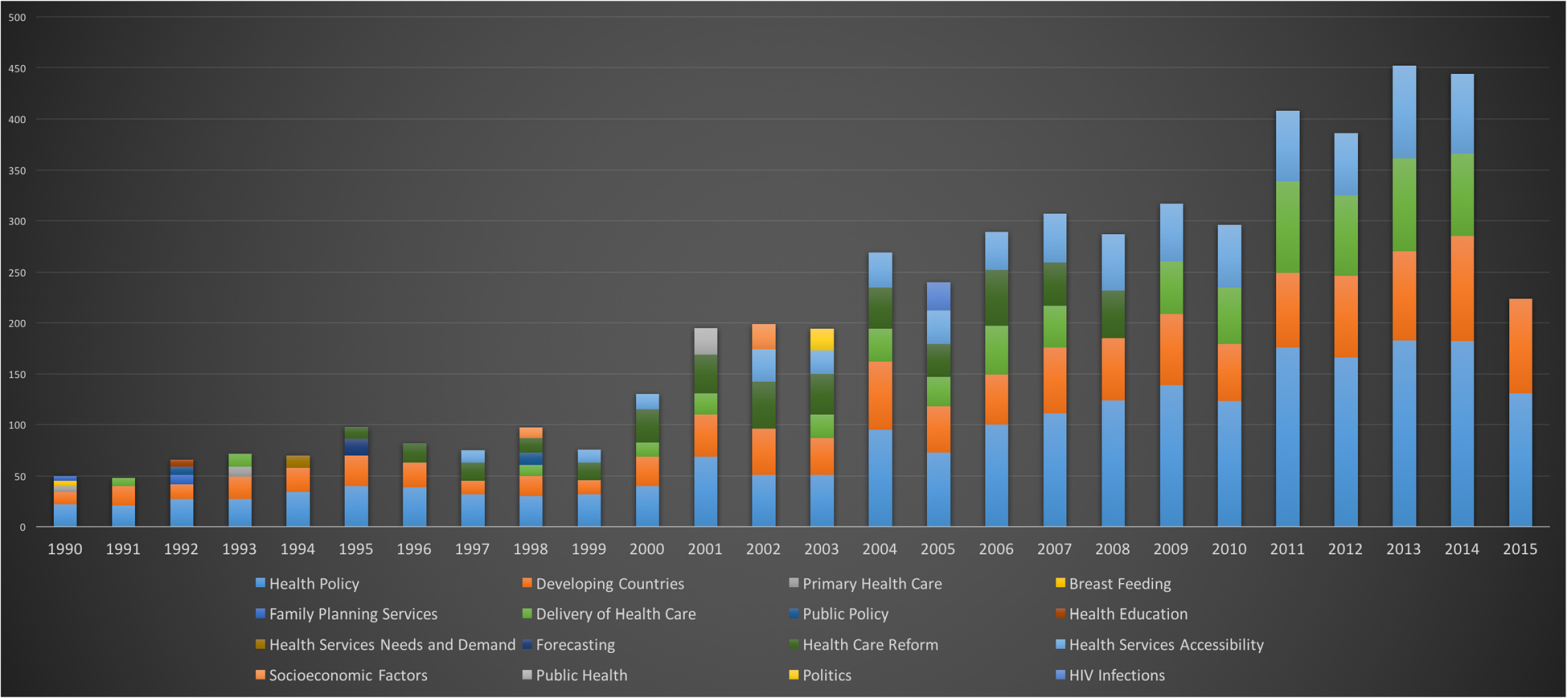
Top MeSH terms assigned to health policy and systems research + low- and middle-income country topic publications, excluding demographics and geography, per year

By removing the MeSH terms limited to sex, age, demographics and specific countries, the terms remaining provide insight on the HPSR topics of interest over time. ‘Health Policy’ and ‘Developing Countries’ are present in all years from 1990 to 2015 (Fig. 14). In the middle of the graph, from 1995 to 2008, ‘Health Care Reform’ was a prominent issue. ‘Health Services Accessibility’ has progressively increased in prominence since the late 1990s. While, ‘Delivery of Health Care’ saw a slow start in the 1990s, but significantly increased in prominence since 2000, to represent a major share of the HPSR MeSH topics since the mid-2000s.

## Discussion

This analysis has primarily examined HPSR using a high-level keyword search strategy, allowing the trends to organically emerge from countries, regions and MeSH terms over time. It is worth noting that, occasionally, MeSH terms are also added/removed based on new research, social relevance and/or political policies, this is specifically evident given addition of the term ‘Health Care Reform’ in 1994.

Historically, analysis incorporated the six BBs, but there are some noteworthy limitations to using the six BBs to analyse HPSR. The current BBs are somewhat arbitrary and these themes have evolved since their initial introduction. In addition, there are inherent overlaps that make them impossible to disentangle and there is no apparent benefit to the discipline in attempting to do so.

Each of the individual BBs are in a very different state of definition. The methods and resulting keywords of published bibliometric strategies were reviewed [13, 15, 18, 19]. The results varied greatly and some studies only defined HPSR in terms of the BB, counterintuitively leaving out ‘health policy’ publications entirely. While some BBs were extensively defined in terms of quantity of keywords, others were very limited and apparently under- or miss-represented by the MeSH terms used (i.e. Information and Evidence/Research was described as ‘Information Systems’ and defined using MeSH terms related to patient/drug records). Few strategies cited the sources from which keywords were drawn, and therefore verification, rationale and limits of the inclusion criteria were difficult to determine. Clinical studies were not explicitly excluded, as per PubMed’s prescribed filters (Additional file 1) [20].

It is difficult to assess whether LMIC authors with multiple affiliations might prefer indicating either their LMIC or HIC affiliation. This could misrepresent the distribution of authors by country classification. To better assess this, we would require knowledge about how many LMIC authors have dual affiliation (both LMIC and HIC) and, among these, how many would opt to indicate their HIC affiliation when writing about and for LMICs. In addition, there is a possibility that LMICs are over-represented in unindexed journals due to language barriers and/or restrictions by National Library of Medicine, etc. Regardless, a database is required, some are indexed and some are not. Unfortunately, there is no way to know what proportion this might represent, whether it is ubiquitous across all income regions or whether it is significant.

In general, bibliometric analysis examines the frequency of publication over time. Co-authorship and citation analysis are an extension of this and are best understood using network analysis. We did not include a list of most-frequently published authors so as to avoid singling out individuals.

## Conclusions

Two decades ago, participation in HPSR by LMICs was low and level. The Alliance for Health Policy and Systems Research began its work in the early 2000s. In looking back via bibliometric analysis, there is a correlation between the timing of specific initiatives resulting from major meetings or reports and increases in the published literature on HPSR that follow. Around the millennium, the momentum from global initiatives focused around HPSR had shifted the knowledge production, interest in and participation by LMICs.

As the Alliance celebrates 20 years of a milestone meeting on HPSR, the momentum for HPSR continues to grow. The progressive advocacy for meaningful participation contributed support for the exponential increase in LMIC authors publishing in the life and biomedical sciences. This increased regional capacity continues to facilitate growth in the literature published on topics relevant to LMICs, such that it has been increasingly outpacing PubMed as a whole (as seen in figures showing normalised slopes). To date, the increased participation shows no sign of slowing down. As this evolution continues, synergies and collaboration lead to a new level of sustained capacity for the individuals, institutions and regions.

The knowledge gained since the introduction of HPSR two decades ago solidified the necessity of using a complexity lens to study health systems as a complex problem. There are many interacting factors that affect and influence each other in different ways over time. Further analysis of the abovementioned synergies would benefit from network analysis.

There is another great opportunity to refine the functional components used to define HPSR. MeSH terms and keyword trends demonstrate the evolution and emergence of relevant issues over time. These trends help clarify where and how to provide additional support and will highlight regions that would benefit from synergies and capacity-building efforts. Understanding and defining HPSR is the necessary foundation for robust analysis of the discipline. Therefore, future work should involve strengthening our understanding, clarifying the scope and definitions for each of the themes (i.e. BBs) used to measure HPSR. A collaborative process to descriptively and methodologically define the inclusive and exclusive criteria would greatly benefit future analysis of the field.

As we look forward toward achieving the SDGs, the outcome of this analysis may be indicative of the positive effect of the ongoing effort to ensure increased funding, institutional capacity-building and knowledge production to continue to support vulnerable populations and resource-constrained settings.

## Additional file

Additional file 1: Supplementary Material. (DOCX 111 kb)

## Abbreviations

BBs: Building blocks
HICs: High-income countries
HPSR: Lealth policy and systems research
LICs: Low-income countries
LMICs: Low- or middle- income countries
Lower-MICs: Lower middle-income countries
MeSH: Medical subject headings
SDGs: Sustainable development goals
Upper-MICs: Upper middle-income countries
