# Supplementary material for: Increasing health policy and systems research capacity in low- and middle-income countries: results from a bibliometric analysis
Source: Health Res Policy Syst. 2017 Jul 28;15:64. doi: 10.1186/s12961-017-0229-1 (PMC5534120; doi:10.1186/s12961-017-0229-1)
Supplement: Supplementary file 1 — Supplementary Material. (DOCX 111 kb) [file 12961_2017_229_MOESM1_ESM.docx]

# Additional file 1

## Databases

To understand the expanding scope of HPSR, several hundred databases within the University of British Columbia (UBC) library databases were scanned for relevance, appropriateness and availability.[^[[1]](#endnote-1)^] The "UBC Catalogue" provides worldwide coverage of electronic resources and greater access to more databases and resources than would be publicly available. This collection includes both indexed (i.e. Web of Science) and non-indexed journals (i.e. Google Scholar).

Using the UBC library guide and in consultation with a librarian, key databases were further identified by searching for traditional disciplines such as mathematics, biology and economics as well as collaborative interdisciplinary fields such as public policy, global health and international relations. Obviously irrelevant databases, such as fine arts and forestry, were excluded; as were duplicate databases, such as MedLine, who’s publications were duplicated within PubMed.

For a recent 10-year period (2004-2013), 11 databases demonstrated an intellectual contribution to the field of HPSR, under traditional and the emerging subject areas. Since many of these databases are not traditionally related to health policy, a preliminary review was conducted of each database using high-level keywords. The highest-level search possible was used to identify the proportion of potentially relevant papers.


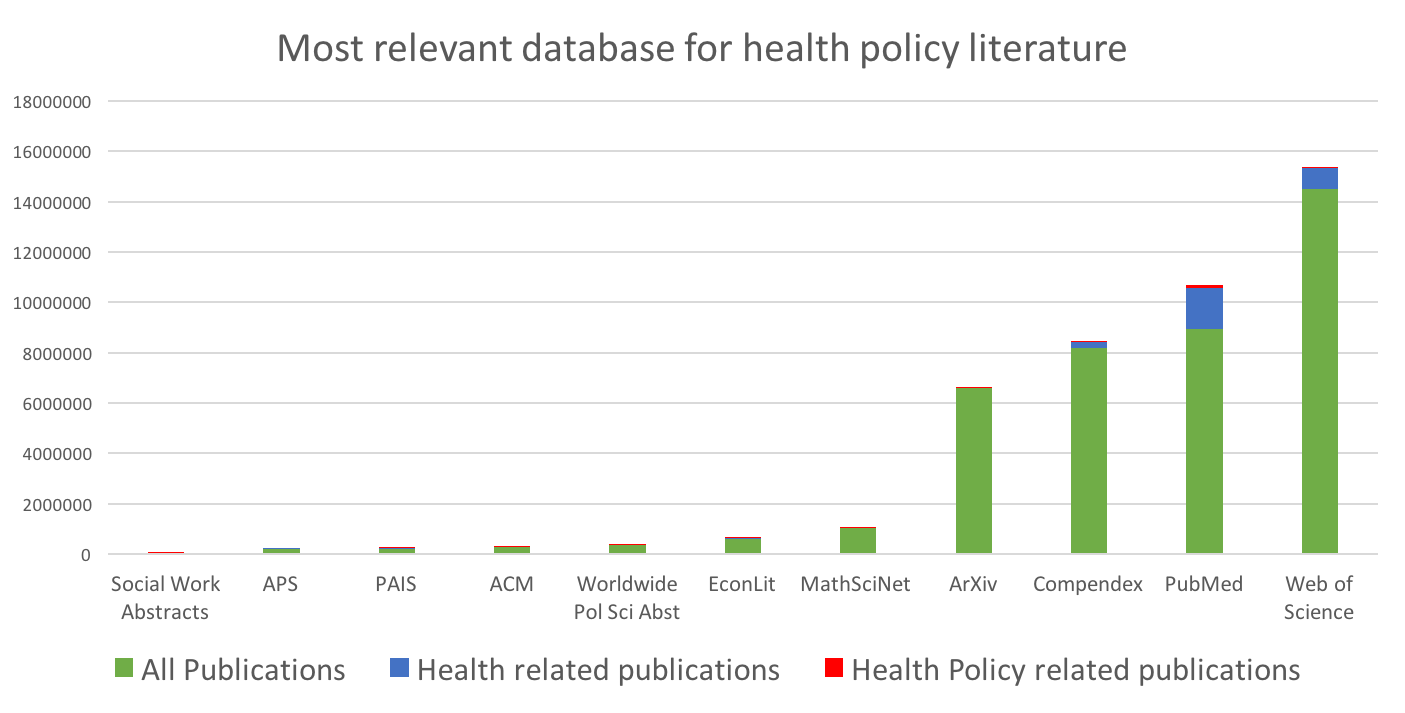


Figure 1: Most relevant database(s) for “health policy” literature

In some cases, the percentage of relevant publications is quite high but the absolute number is low. 46% (n=1128) of papers are related to *health* within the Social Work Abstracts database, similarly the proportion of *health policy* related publications is highest in a Public Affairs (PAIS) database at 13.7% (n=29641).

In terms of absolute numbers, Web of Science is the largest database and is about 50% larger than PubMed, which is the next largest database in size. Yet, PubMed has twice (n=112786) as many papers as Web of Science that are relevant to *health policy*. PubMed is also a free search engine accessing primarily the MEDLINE database of references and abstracts on life and health sciences, and biomedical topics. For these reasons, PubMed was the database selected to conduct the bibliometric analysis of health policy and systems research.

## Country Classification

All Countries as Classified by the World Bank for the 2016 Fiscal Year.

There are 135 LIC, LMIC and UMIC (generally referred to as any LMIC), and 80 HIC identified by the World Bank for the fiscal year 2016 listed in the first 4 columns. The fifth column includes synonyms that are also used in all search strategies including any low-, or middle-income countries. The inclusion of these terms captures papers that may refer to developing countries more generally as a main topic (in the title and or abstract) without listing the name of the country explicitly.

Table 1: List of LMIC and synonyms

| LIC n=31 | LowerMIC n=51 | UpperMIC n=53 | HIC n=80 | Synonyms used for Developing Countries  [dev count] |
| --- | --- | --- | --- | --- |
| Afghanistan | Armenia | Albania | Andorra | developing countr* |
| Benin | Bangladesh | Algeria | Antigua and Barbuda | developing nation* |
| Burkina Faso | Bhutan | American Samoa | Argentina | middle income countr* |
| Burundi | Bolivia | Angola | Aruba | under developed countr* |
| Cambodia | Cabo Verde | Azerbaijan | Australia | LMIC* |
| Central African Republic | Cameroon | Belarus | Austria |  |
| Chad | Congo, Rep. | Belize | Bahamas, The |  |
| Comoros | Côte d'Ivoire | Bosnia and Herzegovina | Bahrain |  |
| Congo, Dem. Rep | Djibouti | Botswana | Barbados |  |
| Eritrea | Egypt, Arab Rep. | Brazil | Belgium |  |
| Ethiopia | El Salvador | Bulgaria | Bermuda |  |
| Gambia, The | Georgia | China | Brunei Darussalam |  |
| Guinea | Ghana | Colombia | Canada |  |
| Guinea-Bisau | Guatemala | Costa Rica | Cayman Islands |  |
| Haiti | Guyana | Cuba | Channel Islands |  |
| Korea, Dem Rep. | Honduras | Dominica | Chile |  |
| Liberia | India | Dominican Republic | Croatia |  |
| Madagascar | Indonesia | Ecuador | Curaçao |  |
| Malawi | Kenya | Fiji | Cyprus |  |
| Mali | Kiribati | Gabon | Czech Republic |  |
| Mozambique | Kosovo | Grenada | Denmark |  |
| Nepal | Kyrgyz Republic | Iran, Islamic Rep. | Estonia |  |
| Niger | Lao PDR | Iraq | Equatorial Guinea |  |
| Rwanda | Lesotho | Jamaica | Faeroe Islands |  |
| Sierra Leone | Mauritania | Jordan | Finland |  |
| Somalia | Micronesia, Fed. Sts. | Kazakhstan | France |  |
| South Sudan | Moldova | Lebanon | French Polynesia |  |
| Tanzania | Morocco | Libya | Germany |  |
| Togo | Myanmar | Macedonia, FYR | Greece |  |
| Uganda | Nicaragua | Malaysia | Greenland |  |
| Zimbabwe | Nigeria | Maldives | Guam |  |
|  | Pakistan | Marshall Islands | Hong Kong SAR, China |  |
|  | Papua New Guinea | Mauritius | Hungary |  |
|  | Philippines | Mexico | Iceland |  |
|  | Samoa | Mongolia | Ireland |  |
|  | São Tomé and Principe | Montenegro | Isle of Man |  |
|  | Senegal | Namibia | Israel |  |
|  | Solomon Islands | Palau | Italy |  |
|  | Sri Lanka | Panama | Japan |  |
|  | Sudan | Paraguay | Korea, Rep. |  |
|  | Swaziland | Peru | Kuwait |  |
|  | Syrian Arab Republic | Romania | Latvia |  |
|  | Tajikistan | Serbia | Liechtenstein |  |
|  | Timor-Leste | South Africa | Lithuania |  |
|  | Ukraine | St. Lucia | Luxembourg |  |
|  | Uzbekistan | St. Vincent and the Grenadines | Macao SAR, China |  |
|  | Vanuatu | Suriname | Malta |  |
|  | Vietnam | Thailand | Monaco |  |
|  | West Bank and Gaza | Tonga | Netherlands |  |
|  | Yemen, Rep. | Tunisia | New Caledonia |  |
|  | Zambia | Turkey | New Zealand |  |
|  |  | Turkmenistan | Northern Mariana Islands |  |
|  |  | Tuvalu | Norway |  |
|  |  |  | Oman |  |
|  |  |  | Poland |  |
|  |  |  | Portugal |  |
|  |  |  | Puerto Rico |  |
|  |  |  | Qatar |  |
|  |  |  | Russian Federation |  |
|  |  |  | San Marino |  |
|  |  |  | Saudi Arabia |  |
|  |  |  | Seychelles |  |
|  |  |  | Singapore |  |
|  |  |  | Sint Maarten (Dutch part) |  |
|  |  |  | Slovak Republic |  |
|  |  |  | Slovenia |  |
|  |  |  | Spain |  |
|  |  |  | St. Kitts and Nevis |  |
|  |  |  | St. Martin (French part) |  |
|  |  |  | Sweden |  |
|  |  |  | Switzerland |  |
|  |  |  | Taiwan, China |  |
|  |  |  | Trinidad and Tobago |  |
|  |  |  | Turks and Caicos Islands |  |
|  |  |  | United Arab Emirates |  |
|  |  |  | United Kingdom |  |
|  |  |  | United States |  |
|  |  |  | Uruguay |  |
|  |  |  | Venezuela, RB |  |
|  |  |  | Virgin Islands (U.S.) |  |

Table 2: Classification of Countries

|  | Number of Countries | GNI per capita (2015) |
| --- | --- | --- |
| Low-income economies (LIC) | 31 | >$1,045 |
| Lower-middle-income economies (LowerMIC)* | 51 | $1,045 - $12,746 |
| Upper-middle-income economies (UpperMIC)* | 53 |  |
| High-income economies (HIC) | 80 | $12,746< |

## Mutual Exclusivity of Countries

To ensure certain countries (e.g., Congo; or North vs. South Korea) remained mutually exclusive to their respective country income classification, specific search criteria were designed. For example, a search for the Republic of Congo, also unintentionally yields all papers in the Democratic Republic of Congo. To remedy this and attempt to achieve mutual exclusivity, Republic of Congo uses the search terms: (((Republic of the Congo) OR République du Congo) OR Congo Republic) OR West Congo), while the Democratic Republic of Congo uses: ((Dem* Rep* Congo) OR Democratic Republic of Congo) OR Zaire). Georgia was yielding disproportionately high results until it was appropriately limited by combining it with several major cities from that country and excluding the city Atlanta, Georgia USA.

The tables below demonstrate a variety of search strategies tested. The results are a basic search in PubMed excluding all other criteria.

| **Dem Rep of Congo (LIC)** |  |
| --- | --- |
| Dem* Rep* Congo[Affiliation] | 9 |
| Democratic Republic of Congo[Affiliation] | 286 |
| Zaire[Affiliation] | 237 |
| ((Dem* Rep* Congo[Affiliation]) OR Democratic Republic of Congo[Affiliation]) OR Zaire[Affiliation] | **531** |
| **Rep of Congo (LMIC)** |  |
| Republic of the Congo[Affiliation] | 186 |
| République du Congo[Affiliation] | 26 |
| Congo Republic[Affiliation] | 1 |
| West Congo[Affiliation] | 39 |
| (((Republic of the Congo[Affiliation]) OR République du Congo[Affiliation]) OR Congo Republic[Affiliation]) OR West Congo[Affiliation] | **242** |

| **“South Korea” (HIC)** |  |
| --- | --- |
| Korea[Affiliation] | 244779 |
| South Korea[Affiliation] | 44209 |
| Rep* Korea[Affiliation] | 7226 |
| Republic of Korea[Affiliation] | 64097 |
| ((South Korea[Affiliation]) OR Rep* Korea[Affiliation]) OR Republic of Korea[Affiliation]) | **111880** |
| **“North Korea” (LIC)** |  |
| Korea, Dem*[Affiliation] | 80 |
| Dem* Korea[Affiliation] | 27 |
| North Korea[Affiliation] | 5 |
| ((Korea, Dem*[Affiliation]) OR Dem* Korea[Affiliation]) OR North Korea[Affiliation]) | **110** |

| **Georgia (LowerMIC)** |  |
| --- | --- |
| Atlanta[Affiliation] | 89439 |
| Georgia[Affiliation] | 84965 |
| Gori[Affiliation] | 170 |
| Sukhumi[Affiliation] | 17 |
| Batumi[Affiliation] | 21 |
| Tbilisi[Affiliation] | 2084 |
| Kutaisi[Affiliation] | 23 |
| ((((Kutaisi[Affiliation]) OR Tbilisi[Affiliation]) OR Batumi[Affiliation]) OR Sukhumi[Affiliation]) OR Gori[Affiliation] | 2309 |
| (Georgia[Affiliation]) AND (((((Kutaisi[Affiliation]) OR Tbilisi[Affiliation]) OR Batumi[Affiliation]) OR Sukhumi[Affiliation]) OR Gori[Affiliation]) | 1832 |
| (((Georgia[Affiliation]) AND (((((Kutaisi[Affiliation]) OR Tbilisi[Affiliation]) OR Batumi[Affiliation]) OR Sukhumi[Affiliation]) OR Gori[Affiliation]))) NOT Atlanta[Affiliation] | **1795** |

# Building Blocks

Health policy and systems research focuses primarily upon policies, organizations and programs, but does not address clinical management of patients or basic scientific research (for example into cell or molecular structures).[^[[2]](#endnote-2)^] In the literature, disciplinary *inclusion* within a building block can be broad while exclusion is more well-defined, yet in practice the boundaries are often blurred. An approach to understanding the field of HPSR is to consider each component to be analyzed. At any given time, each of the 6 building blocks is in different states of development and definition. Given this reality, defining some building blocks is more challenging than others.

Table 3: Health System Building Block Taxonomy

| Terminology as per *WHO “Everybody’s business”* (2007)^[[3]](#endnote-3)^ | Terminology as per *WHO “Monitoring the building blocks”* (2010)^[[4]](#endnote-4)^ | Terminology as per *WHO “Operational Manual” (*2010)^[[5]](#endnote-5)^ | Terminology as per Adam *et al* (2011)^[[6]](#endnote-6)^ | Terminology as per WHO “Health Systems Framework” (2015)^[[7]](#endnote-7)^ | Terminology as per *WHO Alliance “Bibliometric Analysis of HPSR”* (2015)^[[8]](#endnote-8)^ |
| --- | --- | --- | --- | --- | --- |
| Financing | Health Financing | Health Financing | Health Financing | Health Care Financing | Health Financing |
| Health Workforce | Health Workforce | Health Workforce | Human Resources | Health Care Workforce | Health Workforce |
| Information | Health Information | Information and Evidence | Information Systems | Information and Research | Information and Evidence/  Research  (Info & Evid/Res) |
| Leadership and Governance | Leadership and Governance | Leadership and Governance | Governance (and Leadership) | Leadership/  Governance | Leadership and Governance  (Lead & Gov) |
| Medical Products, Vaccines & Technologies | Essential Medicine | Medical Products and Technologies | Medicines | Medical Products, Technologies | Medical Products and Technologies (Med Prod & Tech) |
| Service Delivery | Health Service Delivery | Service Delivery | Service Delivery | Service Delivery | Service Delivery |

Given this is the foundation of HPSR, it is imperative to further refine definitions of each building block if this method is to be used for future analysis. It is important to not only identify relevant key terms, but also clarify appropriate exclusion criteria and continue this process iteratively until the resulting papers appropriately represent each discipline as they were intended.

While attempts have been made to define HPSR by building block,[^[[9]](#endnote-9)^ 6] the name of each building block has changed over the years and therefore carries a different meaning/context depending on the publication. [3 4 5 6 7]

The *naming* of each building block itself is inconsistent across the literature. For example, the building block we refer to as *Information and Evidence/Research* is sometimes referred to as “Information Systems”, and has been defined using only MeSH terms related to patient/drug records.[6] However, this likely underrepresents the intended scope of this building block.

There are inherent overlaps that make it impossible to disentangle the 6 building blocks. In addition, there is no apparent benefit to the discipline in attempting to do so. For this manuscript, we have used high-level definitions for delineation by building block to maintain inclusivity.

Table 4: Syntax for high-level definitions of building blocks

| Building Block (BB) | Abbreviations | Building Block Syntax |
| --- | --- | --- |
| Health Financing | Finance | financ* |
| Health Workforce | HR | (health workforce) OR human resource* |
| Information and Evidence/Research | Info & Evid/Res | {(research) OR evidence} OR (health information) |
| Leadership and Governance | Lead & Gov | leadership OR governance |
| Medical Products and Technologies | Med Prod & Tech | (medical products) OR technolog* |
| Service Delivery | Serv Del | service delivery |

# Clinical Queries Filters

PubMed has a well-defined set of terms combined with Boolean operators that strategically identify clinical and medical genetic research.[^[[10]](#endnote-10)^] This entire set of MeSH terms is excluded from the initial high-level search strategy to result in publication relevant to HPSR.

## Clinical Queries using Research Methodology Filters

| **Category** | **PubMed Equivalent** |
| --- | --- |
| Therapy | ((clinical[Title/Abstract] AND trial[Title/Abstract]) OR clinical trials as topic[MeSH Terms] OR clinical trial[Publication Type] OR random*[Title/Abstract] OR random allocation[MeSH Terms] OR therapeutic use[MeSH Subheading]) |
|  | (randomized controlled trial[Publication Type] OR (randomized[Title/Abstract] AND controlled[Title/Abstract] AND trial[Title/Abstract])) |
| Diagnosis | (sensitiv*[Title/Abstract] OR sensitivity and specificity[MeSH Terms] OR diagnose[Title/Abstract] OR diagnosed[Title/Abstract] OR diagnoses[Title/Abstract] OR diagnosing[Title/Abstract] OR diagnosis[Title/Abstract] OR diagnostic[Title/Abstract] OR diagnosis[MeSH:noexp] OR diagnostic * [MeSH:noexp] OR diagnosis, differential[MeSH:noexp] OR diagnosis[Subheading:noexp]) |
|  | (specificity[Title/Abstract]) |
| Etiology | (risk*[Title/Abstract] OR risk*[MeSH:noexp] OR risk *[MeSH:noexp] OR cohort studies[MeSH Terms] OR group[Text Word] OR groups[Text Word] OR grouped [Text Word]) |
|  | ((relative[Title/Abstract] AND risk*[Title/Abstract]) OR (relative risk[Text Word]) OR risks[Text Word] OR cohort studies[MeSH:noexp] OR (cohort[Title/Abstract] AND study[Title/Abstract]) OR (cohort[Title/Abstract] AND studies[Title/Abstract])) |
| Prognosis | (incidence[MeSH:noexp] OR mortality[MeSH Terms] OR follow up studies[MeSH:noexp] OR prognos*[Text Word] OR predict*[Text Word] OR course*[Text Word]) |
|  | (prognos*[Title/Abstract] OR (first[Title/Abstract] AND episode[Title/Abstract]) OR cohort[Title/Abstract]) |
| Clinical prediction guides | (predict*[tiab] OR predictive value of tests[mh] OR score[tiab] OR scores[tiab] OR scoring system[tiab] OR scoring systems[tiab] OR observ*[tiab] OR observer variation[mh]) |
|  | (validation[tiab] OR validate[tiab]) |

**Medical Genetics Search Filters**

| **Category** | **PubMed Equivalent** |
| --- | --- |
| Diagnosis | (Diagnosis AND Genetics) |
| Differential Diagnosis | (Differential Diagnosis[MeSH] OR Differential Diagnosis[Text Word] AND Genetics) |
| Clinical Description | (Natural History OR Mortality OR Phenotype OR Prevalence OR Penetrance AND Genetics) |
| Management | (therapy[Subheading] OR treatment[Text Word] OR treatment outcome OR investigational therapies AND Genetics) |
| Genetic Counseling | (Genetic Counseling OR Inheritance pattern AND genetics) |
| Molecular Genetics | (Medical Genetics OR genotype OR genetics[Subheading] AND genetics) |
| Genetic Testing | (DNA Mutational Analysis OR Laboratory techniques and procedures OR Genetic Markers OR diagnosis OR testing OR test OR screening OR mutagenicity tests OR genetic techniques OR molecular diagnostic techniques AND genetics) |

# Evolution of MeSH Terms and Keywords Over time

This manuscript examines primarily MeSH terms because the vast majority (approximately 6/7 papers) do not have any author assigned keywords from 2001-2011. It is unclear why this is the case but possibly an indication that PubMed may not have require that field during that period. Despite this shortcoming, a comparison demonstrates some important differences between these categories and the potential implication when selecting one over the other as evidence support.

Table 5 below shows the top MeSH terms and author assigned keywords for the same group of publication (n=7009) during from 1990-2015. The ranked list demonstrates the difference between how the standardized MeSH assignment compares to the author’s characterization the publication. MeSH terms identify the species in every paper, the gender and age group of the population understudy are also given priority. Conversely, author assigned keywords focus on the topic of the paper, with little emphasis demographic information unless it pertains to the socio-economic status as is relevant to LMIC. **This list is significant as it is derived from terms within a high-level keyword search of HPSR literature.** The subsequent graphs (figure??) demonstrate the relevant topics over time. Through these changes over time, the influence and support (by Alliance for HPSR?) for important issues can be observed.

Table 5: A Comparison of most frequently assigned Keywords and MeSH terms within the HPSR literature with a topic relevant to LMIC.

| MeSH | # | Keywords | # |
| --- | --- | --- | --- |
| Humans | 7009 | Developing Countries | 192 |
| Female | 2226 | Health | 134 |
| Health Policy | 2120 | Delivery of Health Care | 97 |
| Male | 1594 | Africa | 89 |
| Adult | 1305 | Economic Factors | 86 |
| Developing Countries | 1197 | Health Services | 79 |
| Delivery of Health Care | 875 | Organization And Administration | 74 |
| Health Services Accessibility | 851 | Population | 74 |
| Middle Aged | 844 | Policy | 72 |
| Health Care Reform | 809 | Primary Health Care | 70 |
| Adolescent | 774 | Demographic Factors | 66 |
| China | 660 | Africa South Of The Sahara | 62 |
| Child | 624 | Health Care and Public Health | 61 |
| Brazil | 596 | Asia | 60 |
| Socioeconomic Factors | 585 | Programs | 53 |
| Public Health | 485 | Family Planning | 51 |
| Surveys and Questionnaires | 469 | Diseases | 49 |
| India | 467 | Public Health | 43 |
| National Health Programs | 448 | India | 41 |
| Young Adult | 444 | Research Methodology | 41 |
| Pregnancy | 428 | English Speaking Africa | 40 |
| Aged | 426 | Research Report | 40 |
| HIV Infections | 422 | Population Characteristics | 37 |
| Cross-Sectional Studies | 418 | Population Dynamics | 37 |
| Public Policy | 414 | Social Policy | 36 |
| Health Promotion | 412 | Socioeconomic Factors | 35 |
| Health Services Needs and Demand | 412 | Developed Countries | 31 |
| International Cooperation | 392 | Health Policy | 31 |
| South Africa | 391 | South Africa | 30 |
| Child, Preschool | 384 | Contraception | 28 |
| Politics | 383 | Financial Activities | 28 |
| Policy Making | 371 | Organizations | 27 |
| Primary Health Care | 371 | Western Africa | 27 |
| Global Health | 360 | Nutrition | 26 |
| Infant | 353 | Program Evaluation | 26 |

# References

1. University of British Columbia Library Indexes & Databases. <http://resources.library.ubc.ca/>. Accessed 8 August 2016. [↑](#endnote-ref-1)
2. Bennett S. Briefing Note 1: What is Health Policy and Systems Research and why does it matter? 2007. World Health Organization, Alliance for Health Policy and Systems Research. <http://www.who.int/alliance-hpsr/resources/Alliance%20HPSR%20-%20Briefing%20Note%201.pdf>. Accessed 8 August 2016. [↑](#endnote-ref-2)
3. World Health Organization. Everybody business: strengthening health systems to improve health outcomes: WHO’s framework for action. World Health Organization 2007. <http://www.who.int/healthsystems/strategy/everybodys_business.pdf>. Accessed 10 September 2015. [↑](#endnote-ref-3)
4. World Health Organization. Monitoring the Building Blocks of Health Systems: A Handbook of Indicators and their Measurement Strategies. 2010. <http://www.who.int/healthinfo/systems/WHO_MBHSS_2010_full_web.pdf> Accessed 17 January 2017. [↑](#endnote-ref-4)
5. Uneke CJ, Ezeoha AA, Ndukwe CD, Oyibo PG, Onwe F, Igbinedion EB, Chukwu PN. Operational Manual for Strengthening Institutional Capacity to Employ Evidence in Health Policymaking for Developing Countries: The Nigeria Experience. World Health Organization; 2010. <http://www.who.int/alliance-hpsr/projects/alliancehpsr_opmanualstrengtheninginstitutionalcapacitynigeria.pdf>. Accessed 17 January 2017. [↑](#endnote-ref-5)
6. Adam T, Ahmad S, Bigdeli M, Ghaffar A, Røttingen J. Trends in Health Policy and Systems Research over the Past Decade: Still Too Little Capacity in Low-Income Countries. PLoS One. 2015; DOI:

   [10.1371/journal.pone.0027263](https://dx.doi.org/10.1371/journal.pone.0027263) [↑](#endnote-ref-6)
7. World Health Organization, Western Pacific Region: The WHO Health Systems Framework. <http://www.wpro.who.int/health_services/health_systems_framework/en/>. Accessed 26 August 2015. [↑](#endnote-ref-7)
8. English K, Pourbohloul B. Bibliometric Analysis of HPSR Literature for The Alliance HPSR: Final Report. 2015  [↑](#endnote-ref-8)
9. Yao Q, Chen K, Yao L, et al. Scientometric trends and knowledge maps of global health systems research. Health Research Policy and Systems 2014, 12:26.

   <http://www.health-policy-systems.com/content/12/1/26>. Accessed 28 January 2016. [↑](#endnote-ref-9)
10. National Center for Biotechnology Information, U.S. National Library of Medicine. Clinical Queries Filters. PubMed Help 2015. <http://www.ncbi.nlm.nih.gov/books/NBK3827/#pubmedhelp.Clinical_Queries_Filters>. Accessed 26 October 2016. [↑](#endnote-ref-10)
